# Editorial: Neuroprotection in Synaptic Signalling During Neurological Disorders

**DOI:** 10.3389/fnsyn.2021.746487

**Published:** 2021-09-03

**Authors:** Matilde Otero-Losada, Francisco G. Wandosell, Francisco Capani

**Affiliations:** ^1^Centro de Altos Estudios en Ciencias Humanas y de la Salud, Universidad Abierta Interamericana, Consejo Nacional de Investigaciones Científicas y Técnicas, CAECIHS.UAI-CONICET, Buenos Aires, Argentina; ^2^Centro de Biología Molecular Severo Ochoa, Consejo Superior de Investigaciones Científicas, Universidad Autónoma de Madrid, CSIC-UAM y Centro de Investigación Biomédica en Red de Enfermedades Neurodegenerativas (CIBERNED), Madrid, Spain; ^3^Centro de Investigaciones en Psicología y Psicopedagogía (CIPP), Facultad de Psicología y Psicopedagogía, Pontificia Universidad Católica Argentina (UCA), Buenos Aires, Argentina

**Keywords:** synapse, intracellular pathway, translational medicine, neurodegeneration, pharmacological strategies

Some of the most devastating and costly conditions in the world arise from neurological and neurodegenerative disorders. About one-third of the world's population suffers from neurological diseases, including Parkinson's (PD) and Alzheimer's (AD) diseases, multiple sclerosis, epilepsy, spinal cord injury, and others. Symptoms may show early in childhood or substantially delayed in adolescence or young adulthood. Animal models are powerful tools aiding in understanding neurological pathophysiology towards developing neuroprotective strategies.

So far, there are no well-established treatments for brain repair. Hence, neuroprotection becomes imperative. Synapses pose major targets for finding new neuroprotective agents.

The aim of this topic is to share research addressing neuroprotective strategies for the brain, their possible pathways, and the use of pharmacological analogues. Four reviews are about hypoxia. Two chapters address the molecular bases of cognitive impairment in AD. Biochemical and physiological aspects of neuroprotective agents focusing on synaptic modifications, neuroprotection following perinatal asphyxia, and pharmacological- and genetic-targeting strategies for Alzheimer's and Parkinson's diseases are reviewed.

Synaptoprotection in Perinatal Asphyxia: An Experimental Approach of Herrera et al., a mini review on this obstetrical complication occurring when the oxygen supply to the newborn is interrupted, associated with high morbimortality. Around 25% of PA survivor newborns develop several neurodevelopmental disabilities because of severe synaptic damage. Synaptic dysfunction embodies a putative target for neuroprotective strategies. Over the last years, therapeutic hypothermia, the only treatment available, has shown positive results in the clinic [(Barkhuizen et al., 2017; Herrera et al., 2017); (3)]. Several pharmacological agents are being tested in experimental or clinical trial studies to prevent synaptopathy. Synaptoprotection makes up a promising challenge for reducing incidental neurodevelopmental disorders associated with PA.

The Renin–Angiotensin System Modulates Dopaminergic Neurotransmission: A New Player on the Scene of Kobiec et al. Parkinson's disease (PD), an extrapyramidal neurodegenerative disorder, has become a major health problem, affecting 1% of the world population over 60 years old and 3% of people beyond 80 years. The renin–angiotensin system (RAS), regulating blood pressure and body fluid balance, is also involved in autocrine and paracrine regulation of nigrostriatal dopaminergic synapses (Capani et al., 2009; Labandeira-Garcia et al., 2017; Jackson et al., 2018; Lang and Espay, 2018; Simon et al., 2020). Dopamine depletion, as in PD, increases angiotensin II expression, which stimulates or inhibits dopamine synthesis and is released via angiotensin II type 1 or 2 receptors. Furthermore, the angiotensin II type 1 receptor inhibits D1 receptor activation allosterically. Therefore, the RAS may have an important modulating role in the flow of information from the brain cortex to the basal ganglia.

Nanoliposomes as a Therapeutic Tool for Alzheimer's Disease of Ordóñez-Gutiérrez and Wandosell. This chapter covers promising therapeutic avenues for AD treatment. Nanoliposome therapies for AD, still in clinical trials, are discussed, and nanoliposomes-bound anti-amyloid-beta (Aβ) peptide antibodies are presented. Extracellular Aβ deposits (senile plaques) and intracellular neurofibrillary tangles of hyperphosphorylated Tau protein are two major neuropathological AD hallmarks. Brain and peripheral Aβ levels are in equilibrium. Enhancing peripheral clearance might reduce brain Aβ level, known as the sink effect. Nanoparticles may have difficulty in crossing the blood-brain barrier unless derivatized. Several nanoparticles' derivatives have been proposed, carrying antibodies against Aβ (Carradori et al., 2018).

An Assessment of Melatonin's Therapeutic Value in the Hypoxic-Ischemic Encephalopathy of the Newborn of Cardinali. Hypoxic-ischemic encephalopathy is one of the most frequent causes of brain injury in the newborn. According to the World Health Organisation, for every day in 2015, 16,000 children aged under five died (World Health Organization, 2015). Melatonin has impaired chronic mechanisms of neuronal death. In animal models, and in a limited number of clinical studies, melatonin increased the level of protection developed by hypothermia in newborn asphyxia (Xu et al., 2017). This review summarises therapeutic strategies, assessing the role of melatonin as a potentially relevant therapeutic tool to cover the hypoxia-ischemia (HI) phase and the secondary and tertiary phases following a HI insult.

Fetal Neuroprotective Strategies: Therapeutic Agents and Their Underlying Synaptic Pathways of Elsayed et al. Emerging evidence suggests that melatonin and N-acetyl-L-cysteine (NAC) may also serve as novel putative foetal neuroprotective candidates. Melatonin has important anti-inflammatory and antioxidant properties and is a known mediator of synaptic plasticity and neuronal generation. While NAC acts as an antioxidant and a precursor to glutathione, it also modulates the glutamate system. Glutamate excitotoxicity and dysregulation can induce perinatal preterm brain injury through damage to maturing oligodendrocytes and neurons. The improved drug efficacy and delivery of the dendrimer-bound NAC conjugate provides an opportunity for enhanced pharmacological intervention. Recent literature on the synaptic pathways underlying NAC and melatonin effects is reviewed, discussing the current gaps in knowledge, and proposing future directions for foetal neuroprotection (Dean et al., 2011).

Current therapies for neonatal hypoxic-ischaemic and infection-sensitised hypoxic-ischaemic brain damage of Tetorou et al. Therapeutic hypothermia is the only approved treatment for neonatal HI. However, the number of HI infants needed to treat with hypothermia for one to be saved from death or disability at age of 18–22 months, is ~6–7, highlighting the urge for alternative or additional strategies. The authors discuss the mechanisms of HI injury to the immature brain and the new experimental treatments studied for neonatal HI and infection-sensitised neonatal HI (Nair and Kumar, 2018).

Early Effects of Aβ Oligomers on Dendritic Spine Dynamics and Arborization in Hippocampal Neurons of Ortiz-Sanz et al. Aβ oligomers induce synaptic damage early in Alzheimer's disease. An open question for understanding AD pathology is how soluble Aβ contributes to dendritic spine loss and dendritic simplification, as there are a large number of putative Aβ receptors (Jarosz-Griffiths et al., 2016). The authors examined the acute effects of soluble Aβ42 on spine dynamics, dendritic alteration, and signalling pathways, using hippocampal neurons, and high-resolution imaging followed by algorithm-based evaluation of spine changes and alterations of dendritic arborization. Acute Aβ oligomers increased spine density by mechanisms involving integrin β1 and CaMKII signalling, promoting dendritic complexity in CA1 hippocampal neurons.

Next coming, neuroprotection strategies might point to immunotherapy. The FDA, through an Accelerated Approval Program, has recently approved an anti-amyloid antibody as the first new option in AD treatment. Yet, improved human-akin accurate experimental models are still missing. Intensive genetic manipulation, like the need to introduce three dominant mutations to have an AD-like mice phenotype, makes the model controversial. Besides, many promising treatments in experimental stroke and head and spinal cord injury have failed in clinical trials. The road to identifying successful neuroprotection approaches is still to be walked through research.

## Author Contributions

All authors listed have made a substantial, direct and intellectual contribution to the work, and approved it for publication.

## Conflict of Interest

The authors declare that the research was conducted in the absence of any commercial or financial relationships that could be construed as a potential conflict of interest.

## Publisher's Note

All claims expressed in this article are solely those of the authors and do not necessarily represent those of their affiliated organizations, or those of the publisher, the editors and the reviewers. Any product that may be evaluated in this article, or claim that may be made by its manufacturer, is not guaranteed or endorsed by the publisher.
